# Psychosocial burdens and unmet supportive care needs of partners and relatives of individuals with Li‐Fraumeni syndrome: A mixed‐methods study

**DOI:** 10.1002/jgc4.70148

**Published:** 2025-11-28

**Authors:** Senta Kiermeier, Sarah Schott, Juliane Nees, Christina M. Dutzmann, Farina Silchmüller, Christian P. Kratz, Myriam Keymling, Imad Maatouk

**Affiliations:** ^1^ Chair of Integrated Psychosomatic Medicine and Psychotherapy, Department of Internal Medicine II University Hospital Würzburg Würzburg Germany; ^2^ Department of Gynecology and Obstetrics University Hospital Heidelberg Heidelberg Germany; ^3^ Breast Unit Sankt Elisabeth Hospital Heidelberg Germany; ^4^ Department of Pediatric Hematology and Oncology Hannover Medical School Hannover Germany; ^5^ Department of Radiology German Cancer Research Center (DKFZ) Heidelberg Germany

**Keywords:** hereditary cancer syndrome, Li‐Fraumeni syndrome, partner, supportive care needs, TP53

## Abstract

Li‐Fraumeni syndrome (LFS) is a rare but highly penetrant cancer predisposition syndrome caused by pathogenic variants in the tumor suppressor gene *TP53*. Individuals diagnosed with LFS should adhere to intense surveillance programs for early tumor detection. The literature highlights several psychosocial challenges for this group. However, the scarce and mainly qualitative research on LFS families suggests that people close to individuals with LFS (e.g., partners, spouses, kin, friends) are likely also burdened by this condition. Therefore, the aim of our study was to assess their unmet supportive care needs (uSCN) as well as the psychosocial burdens and challenges they face. For this convergent mixed‐methods study, first, we used validated questionnaires: the Supportive Care Needs Survey for Partners and Caregivers (SCNS P&C) to assess uSCN; the short form of the Fear of Progression questionnaire for partners (FoP‐Q‐SF/P); the distress thermometer (distress of last week on a scale from 0 to 10), and the corresponding problem list. Descriptive statistics were used to analyze quantitative data from a total of 43 participants. The majority reported clinically relevant levels of distress (70%) and fear of progression (56%). With respect to uSCN, “health‐care services and information needs” and “emotional and psychological needs” were the most relevant. “Feelings about death” was the item that was reported as unmet the most (69%). Second, we conducted additional semi‐structured telephone interviews on unmet needs and challenges with 19 of our participants, which we transcribed and analyzed via content analysis. Interviewees reported high involvement in organizing and managing life around LFS, with “emotional and problem‐focused coping” strategies. Our study reveals numerous informational and emotional burdens and uSCN in partners and relatives of individuals with LFS. A familial or systemic approach to genetic counseling and health care may be beneficial for improving the well‐being of individuals who are directly and indirectly affected by LFS.

**What is known about this topic**

The diagnosis of and living with a cancer predisposition syndrome such as Li‐Fraumeni syndrome (LFS) is a physically and mentally challenging condition. LFS is inherited dominantly; therefore, several members of a family are likely affected.

**What this paper adds to the topic**

We provide insights into the challenges faced by those in the social circle of individuals with LFS. Even if not directly affected by the elevated cancer risk, the majority of partners, relatives, and friends revealed clinically relevant levels of fear of progression, distress, and reported several unmet supportive care needs. Therefore, they need to be accounted for during the care of individuals with LFS.

## 1 INTRODUCTION

Li‐Fraumeni syndrome (LFS) is a hereditary cancer syndrome with autosomal dominant inheritance. It is caused by pathogenic germline variants in the gene *TP53*, which codes for the protein p53 that is crucial for tumor suppression. Previous research demonstrates that the type of variant present in the *TP53* gene is relevant for the severity and phenotypic expression of LFS and likely also unappreciated modifying factors under study (Kennedy & Lowe, 2022; Kratz et al., 2021). LFS in general is associated with a highly elevated risk of developing a wide range of tumors across the lifespan, for example, breast cancer, brain cancer, sarcomas, or adrenocortical carcinoma. The possible onset begins in early childhood. There is evidence suggesting that some *TP53* variants particularly predispose individuals to specific tumor types (Kennedy & Lowe, 2022). To improve the survival rate, an intensive surveillance program has been recommended internationally (Kratz et al., 2022; Villani et al., 2011). Individuals with LFS often need to face several challenges: genetic testing, cancer diagnosis and treatment, uncertainty about the future, and scheduling and committing to surveillance. As a result of their condition, they are at risk for developing psychosocial symptoms, for example, elevated levels of anxiety and distress (Gopie et al., 2012; Kiermeier et al., 2024; Rippinger et al., 2020). We also previously identified low health‐related quality of life (HRQOL) in individuals with LFS for both mental and physical aspects of HRQOL, regardless of cancer diagnosis (Kiermeier et al., 2024).

Fear of progression (FoP) refers to the fear of cancer developing, recurring, or progressing in cancer patients or survivors and represents a significant concern in psycho‐oncological care (Herschbach et al., 2010). Considering the degree of uncertainty associated with living with LFS, we previously explored FoP in individuals with LFS and revealed high levels of FoP in the majority of participants, regardless of whether they had a prior actual cancer diagnosis (Kiermeier et al., 2024).

Research indicates that psychological symptoms in cancer caregivers and partners are not only prevalent but also interwoven with patients' experiences, for example, FoP in partners increases with the amount of uncertainty and uncontrollability of their partners' disease (Zimmermann et al., 2011) and significant negative effects, such as anxiety and FoP, on partners of patients with advanced prostate cancer (Sauer et al., 2022). This has also been observed in hereditary cancer syndrome cohorts including LFS, where about 25% of partners exhibited clinically significant distress levels. Moreover, the distress and worries of these partners showed a significant correlation with those of the diagnosed individuals (Lammens et al., 2011). Considering that caregivers also experience a wide range of unmet supportive care needs (uSCN) (Wang et al., 2018), owing to the hereditary nature of LFS and the fact that cancer itself is often referred to as a “we disease,” it is important to look at the social environment of individuals with LFS. In LFS families, in which it is likely that more than one family member is diagnosed, family structures and interactions are often very dynamic (Pantaleao et al., 2020). LFS family members can influence each other in terms of their perceptions of cancer risk and participation in cancer screening, as well as their decision‐making and communication strategies (Huelsnitz et al., 2025; Wilsnack et al., 2021). Given that this influence on (mental) health behavior can be crucial for survival in the context of LFS (Huelsnitz et al., 2025), it is essential to explore in greater detail the burdens and uSCN experienced both within and outside the family of individuals with LFS. This deeper understanding of caregivers is necessary to better support them and the under‐researched LFS population. As previously stated: For individuals with LFS, “family, friends, spouses, and confidantes are especially important” to emotionally cope with their condition (Peters et al., 2016). Such support in emotional coping may be reciprocal, yet adverse consequences have been observed, for instance when emotional experiences are hidden to protect one another, potentially leading to a distressing disconnection in the relationship (Young et al., 2019) or when strategies to support the high‐risk individual are perceived as disrespecting their autonomy (Huelsnitz et al., 2025).

Nevertheless, previous studies on individuals indirectly affected by LFS are scarce, and those studies focus on qualitative data. Although these allow to gain detailed insights into a small number of individual LFS cases, quantitative methods using validated questionnaires are also needed to better classify this unique condition and systematically assess their needs and challenges in comparison to other cohorts. The combination in the form of a convergent mixed‐methods approach may be particularly advantageous for this scarcely studied group (Borle & Austin, 2025; Wainstein et al., 2023). Therefore, we present the first German study that quantitatively and qualitatively assesses spouses, parents, siblings and friends of individuals with LFS. The primary purposes of this study were (1) to characterize the concerns, burdens, and uSCN perceived by the partners, relatives, and friends of individuals with LFS in Germany; (2) to determine the extent of distress, FoP, and health‐related quality of life (HRQOL) of those individuals; and (3) to determine how they cope with LFS‐related challenges. On the basis of our findings, we want to provide supporting data on how to address identified burdens and implement supportive care structures for partners and relatives of individuals with LFS.

## 2 METHODS

### 2.1 Study design and participants

In this convergent mixed‐methods study, we collected both qualitative and quantitative data in close succession. Individuals were eligible for this study if they were 18 years or older, fluent in speaking German, did not have an LFS diagnosis themselves (negative test result or not relevant, e.g., partner or friend), and considered themselves close to a person with LFS. The participation of more than one significant other per individual with LFS was possible. Potentially eligible individuals were approached in three different ways: (1) in person when accompanying individuals with LFS during surveillance appointments at University Hospital Heidelberg and Hannover Medical School (2) at LFS‐specific information events or (3) via a German LFS‐focused Facebook group. If individuals with LFS were present at (1)–(3), we encouraged them by explaining our study aims to share study information with their partners, relatives, and friends. In most cases, the high‐risk individual already took part in our parallel study exploring psychosocial burdens and needs in individuals with LFS (Kiermeier et al., 2024, 2025). Eligible participants were asked whether they wished to participate only in the survey component of the study or also in the interview component. Accordingly, all participants who took part in the interviews had also prior completed the study questionnaires. Participants did not receive any form of compensation for their involvement in the study. Data were collected from March 2020 to June 2021. Qualitative and quantitative data were analyzed separately and then merged.

### 2.2 Quantitative measures and analysis

A survey was prepared and accessible online or via paper/pencil. It included eight investigator‐designed demographic items (e.g., marital status, current work status) and 13 LFS‐related items (e.g., surveillance adherence, degree of kinship, tumor history). The following validated questionnaires were used: We used the German version of the National Comprehensive Cancer Network Distress Thermometer and problem list to assess distress (0 = no distress, 10 = extreme distress), which revealed acceptable sensitivity and specificity in a German validation study. The additional problem list consists of 35 yes/no items such as “insurance,” “sleep,” or “pain” (Mehnert et al., 2006). The German version of the Short Form‐12 Health Survey (SF‐12) (Drixler et al., 2020) was used to assess HRQOL. The SF‐12 results in two scores: physical component of HRQOL (PCS) and mental component of HRQOL (MCS). Raw scores are converted (mean = 50, SD = 10), ranging from 0 (lowest state of HRQOL) to 100 (highest state of HRQOL). The SF‐12 previously revealed acceptable internal consistency (Cronbach's α = 0.72–0.92) (Drixler et al., 2020). We used the 12‐item FoP Questionnaire for Partners – Short Form (FoP‐Q‐SF/P) to assess FoP (Cronbach's α 0.87). The response categories vary between 1 (never) and 5 (very often). Therefore, sum scores range from 12 to 60, higher values indicating higher levels of anxiety (Zimmermann et al., 2011). Furthermore, we used the Supportive Care Needs Survey for Partners and Caregivers (SCNS P&C) as a commonly used questionnaire to investigate uSCN in the past month (Wang et al., 2018). The SCNS P&C consists of 45 items in four domains: (1) health‐care service and information needs, (2) emotional and psychological needs, (3) work and social security needs and (4) communication and family needs. As the German validation study on SCNS P&C suggests, we removed items 18 and 29 from domain analysis (Sklenarova et al., 2015). On a five‐point Likert scale, participants could indicate whether they do not have a need (1), their need is met (2), or if their unmet need is low (3), medium (4) or high (5). Items were transformed (range 0–100) to take different item numbers into account. Internal consistencies across domains have shown to be acceptable (Cronbach's α = 0.95–0.76) (Sklenarova et al., 2015). One participant had to be removed from the SCNS P&C analysis because too many items were missing.

We adapted the wording of SCNS P&C and FoP‐Q‐SF/P if necessary to match our cohort (e.g., “LFS” instead of “cancer”). Reliability analysis with Cronbach's *alpha* revealed satisfactory internal consistency for the questionnaires with adapted wording: α = 0.895 (FoP‐Q‐SF/P), α = 0.929 (SCNS P&C—health‐care service and information needs), α = 0.813 (SCNS P&C—emotional and psychological needs), α = 0.872 (SCNS P&C—work and social security needs), and α = 0.863 (SCNS P&C—communication and family needs).

To ensure comparability within our study, we used the same thresholds to indicate clinically relevant levels of psychological impairment for individuals with LFS and for their partners or relatives (Kiermeier et al., 2024), which are depicted in Table 1. IBM SPSS (Version 29) software was used for descriptive analysis of the quantitative data. The Statement on “Strengthening the Reporting of Observational Studies in Epidemiology” (STROBE) is provided in the Data S1.

**TABLE 1:** Results for quality of life, fear of progression, and distress (*N* = 43).

	Min	Max	Mean	SD	Possible range	Threshold indicating clinical relevance	Above/under threshold, *N* (%)
Physical component score	24.25	60.77	48.82	9.85	0–100	≤45	13 (30.2%)
Mental component score	24.21	63.88	45.99	11.31	0–100	≤47	19 (44.2%)
Fear of progression sum score	15	56	35.58	10.47	12–60	≥34	24 (55.8%)
Distress last week, including today	0	9	5.84	2.49	0–10	≥5	30 (69.8%)

### 2.3 Interview data collection and qualitative data analysis

**FIGURE 1:**
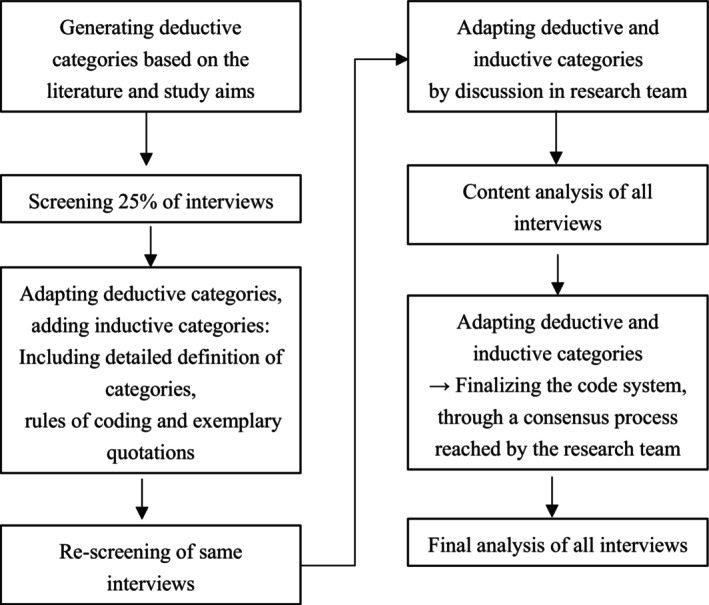
Process of analyzing interview data.

A semi‐structured interview script consisting of 50 mostly open‐ended questions was developed by authors SK (PhD candidate and psychologist), IM (medical expert trained in internal medicine and psychosomatics and clinician scientist—Full Professor), SS (medical expert on LFS, Gynecologic oncologist, clinician scientist—Associate Professor), and JN (board certified gynecologist, clinician scientist) based on their clinical experience with LFS and review of the current literature. We invited participants of the study questionnaire to additionally take part in the study interview. Author SK organized participant recruitment and conducted all semi‐structured interviews by telephone. The interview questions addressed the following topics: perceived benefits and burdens of surveillance, challenges, resources and coping strategies, health systems, and relationship with individuals with LFS. The interviews were audiotaped and transcribed. Author SK analyzed transcribed interviews. First, some deductive categories were derived based on our clinical knowledge of LFS and pre‐existing literature, while inductive categories were then added in the process (Kuckartz, 2018), resulting in an iterative process of comparing and asking questions about the content. Given that the focus of the analysis was on the inductively derived categories, our approach can most appropriately be characterized as an “inductive qualitative content analysis” (Lynch et al., 2024; Wainstein et al., 2023) (Figure 1). In line with our approach of a convergent mixed‐methods study, for data integration of qualitative and quantitative results, we aimed to compare the statistical results with the findings from the qualitative analysis in order to explore the extent to which they converge, complement, or contradict each other (“data comparison” approach). Data were integrated and connected at and after the data analysis level. We did not perform data transformation (Borle & Austin, 2025). Analysis was conducted via MAXQDA® 2021 (VERBI Software). We did not perform inter‐coder reliability; instead, categories and evolving ambiguities were continuously discussed among authors SK, SS, and IM. Recruitment stopped when data saturation was reached in analysis, meaning no more inductive categories were added when screening the interviews. We did not estimate sample size beforehand, as knowledge about caregivers of individuals with LFS is scarce. The final category system was consented by all three. No other authors were involved in qualitative analysis. Transcripts were not returned to participants. The “Consolidated criteria for Reporting Qualitative research” (COREQ) checklist is provided in the Data S1 (Table S1).

## 3 RESULTS

### 3.1 Participant characteristics

A total of 43 individuals participated in the study. Mean age was 38 years (*SD* = 8.73) and 23 (53%) were female. The majority of participants were married (56%) or in a committed relationship (19%). More than half were employed (56%), and the median monthly net household income was €3200. Overall, 42% of participants held a university degree. More than half (65%) of the study participants were partners or spouses, whereas four were friends and one was a sibling. The option “other” was chosen frequently, as it allowed participants to indicate more than one relationship, for example, “sibling and father,” “partner and mother,” “my three children.” Although all participants of this study were not diagnosed with LFS themselves, four study participants were cancer survivors. Ten relatives have experienced their child having cancer and the death of a family member was experienced by four participants. More details on the characteristics of our sample can be found in Nees et al. (2022). For the subcohort of interview participants (*N* = 19), again, the majority were partners (*N* = 10, 52.6%), two were friends (10.5%), one was a sibling (5.3%), and six were “others” (41.6%). The mean age of the interviewees was 40.8 years, and 58% were female. Six (31.6%) interviewees had a child diagnosed with cancer, and two (10.5%) had lost a child due to cancer. One (5.3%) interviewee had lost a parent, and one interviewee had been diagnosed with cancer (no underlying LFS).

### 3.2 Fear of progression, distress, and health‐related quality of life

The descriptive results of the study questionnaires are presented in Table 1: For FoP, the mean score was above the predefined cutoff for dysfunctional FoP, with 56% of participants indicating a clinically elevated level. With a mean score of 5.84 in the NCCN distress thermometer, almost 70% of the participants presented high distress in the previous week. Approximately one‐third of the participants (30.2%) demonstrated low functioning in the physical component score (PCS) of HRQOL, and 44.2% of our sample displayed clinically low levels in the mental component score (MCS).

### 3.3 Supportive care needs and identified problems

The analysis of the SCNS‐P&C‐34 subscales revealed the highest mean score for the subscale of “health‐care services and information needs” (*M* = 35.01, SD = 23.01), followed by “emotional and psychological needs” (*M* = 32.82, SD = 23.00), “work and social security needs” (*M =* 25.68, SD = 24.46), and “communication and family needs” (*M* = 24.05, SD = 27.01), with higher mean scores (range 0–100) indicating more uSCN. The subscale “communication and family needs” was not present among the top 10 unmet needs items (Table 2). The most reported uSCN were “feelings about death” (69%), “feeling confident that all the doctors are talking to each other to coordinate the person with LFS's care” (64.3%), and “ensuring there is an ongoing case manager to coordinate services for the person with LFS” (64.3%). The problems identified by more than 20% of the participants via the distress problem list are displayed in Table 3. “Worry” was the most prominent term (76.7%), followed by “fatigue” (67.4%), “sleep” (60.5%), and “fear” (55.8%).

**TABLE 2:** Prevalence of top unmet supportive care needs of partners and caregivers (*N* = 42).

Rank/item	*N* (%)
1. Feelings about death (E)	29 (69%)
2. Feeling confident that all the doctors are talking to each other to coordinate the person with LFS's^a^ care (H)	27 (64.3%)
3. Ensuring there is an ongoing case manager to coordinate services for the person with LFS^a^ (H)	27 (64.3%)
4. Accessing information about the benefits and side effects of treatments (H)	26 (61.9%)
5. Managing concerns about the cancer coming back (E)	26 (61.9%)
6. Having opportunities to discuss your concerns with the doctors (H)	24 (57.1%)
7. Changes to patient's life/work (W)	24 (57.1%)
8. Understanding the experience of the person with LFS^a^ (E)	23 (54.8%)
9. Obtaining the best medical care for the person with LFS^a^ (H)	23 (54.8%)
10. Financial/government support (W)	23 (54.8%)

Abbreviations: (E), emotional and psychological needs; (H), health‐care services and information needs; (W), work and social security needs.

^a^ Whenever suitable, we adapted the item wording to “LFS” instead of “cancer.”

**TABLE 3:** Results of the most often (>20%) mentioned problems on the distress thermometer problem list.

	*n* (N= 43)	%
Family problems		
Dealing with partner	15	34.9
Emotional problems		
Worry	33	76.7
Fears	24	55.8
Sadness	20	46.5
Depression	12^a^	29.3
Nervousness	14^b^	33.3
Physical problems		
Pain	20	46.5
Fatigue	29	67.4
Sleep	26	60.5
Getting around	18	41.9
Skin dry/itchy	13	30.2
Nose dry/congested	14	32.6

^a^ *N* = 41.

^b^ *N* = 42.

### 3.4 Qualitative results

We conducted 19 interviews, lasting 50 min on average and ranging from 30 to 98 min. With qualitative content analysis, we identified three main categories (I–III) and 10 subcategories (a–j). The categories are defined in Table 4.

**TABLE 4:** Resulting categories of inductive qualitative content analysis.

Main category	Category	Definition	Example
I Psychological needs	(a) Inheritance and genetic testing	Hereditary aspects of LFS: Descriptions on how LFS influenced thoughts on one's own family planning as well as past experiences with cancer in the family	*So sometimes I think to myself, if I had known it before (…), when we had a child, we knew what was going to happen. We knew about the risk that the kid was going to get it. We knew it. We had to decide. We decided to have the child, but I could not have imagined at that time that it would be so hard.*
	(b) Cancer diagnosis and loss	Interviewees describing the situation of witnessing been diagnosed with cancer and its treatment	*Yes, it's kind of like (…) déjà vu. The first time, of course, you don't know what to expect. And the second time, everything goes through your head again, everything that happened with the chemo, the late effects, how my wife felt. You can see it all again in front of your eyes*
	(c) Witnessing emotional responses of individuals with LFS	Interviewees describe emotional responses on LFS‐related challenges of individuals with LFS they have witnessed	*Yes, very often very bad. So also really with (sentences like) “I can't do this anymore, I don't want to anymore, I'm going to stop now and go to Switzerland and get an injection there”* (Note: refers to seeking assisted suicide).
	(d) Fear of disease for the individual with LFS, one's own fear and worries regarding the future	Statements on anxiety, fear, and worries related to LFS and cancer, witnessed and experienced	*Simply fear. It is the pure fear that at any time something is somewhere again. And it's certainly a big mental issue, because you can't do anything about the disease, but it's really very much a mental thing.*
	(e) Coping strategies	Interviewees describing how they cope with different challenges caused by LFS	*Grit your teeth and get through it.*
II Health system and information needs	(f) Desire of a central contact point	Experiences with health‐care providers with special focus on how they should be organized and interact with each other	*We had a time where we were flying from one doctor to another and no one really knew what to do with it. During that time, we were pretty scared.*
	(g) Need of psychosocial support	Attitude toward and experience with psychosocial support	*Often, I put up a brave front. And at some point the emotional barrel is brimmed. So, if you then have no one to whom you can tell it, of course it is always a bit difficult.*
	(h) Involvement in organizational aspects	Interviewees describing their involvement in organizing LFS in daily life	*So, it's like everything always goes through me and everyone also talks to me. And I just think that's good.*
III Communication needs	(i) Impact of LFS on daily communication	Interviewees describing their communication within their social life, how (much) it is influenced by LFS topics and how they wished communication would be. Additionally, if and why they do (not) communicate with other persons with LFS	*Since we know that this gene is in the family, above all those who have the gene, there is always such a depressed mood, again and again.*
	(j) Communicating with physicians	Examples and expectations on how doctors (should) communicate with interviewees	*“That's not my area of expertise, goodbye, you'll have to take care of that yourself.” I felt completely let down.*

#### 3.4.1 Main category I: psychological needs

Psychological needs are influenced by the hereditary aspects of LFS and its repercussions (Table 4a). As the majority of our participants were partners or spouses of an individual with LFS, the struggle regarding their wish to have children was often mentioned as a psychological burden. One individual was worried about their grandchildren with LFS “not much longer and then they will also have to deal with the issue” (ID 7). When children inherited LFS from one parent, most reported that the knowledge of passing it on was burdening for the individual with LFS and the whole family (“My father was just screaming when he realized that he passed it on to his daughter,” ID 65). Another psychological challenge was the process of genetic testing. This was reported as a straining phase, whether it was their own testing or of their loved ones (“Please, please, please, let it just be negative,” ID 10). Additionally, ethical and legal challenges in testing children were mentioned (“It turned out to be very complicated to test children for this, due to the approvals required for doctors to even take blood samples,” ID 16). Moreover, the participants reported a lack of information on pre‐implantation genetic testing. One woman reported how she disapproved of the decision of her sibling with LFS not testing her children, which led to a disruption of their sibling relationship.

Witnessing a cancer diagnosis or a result that required further testing was another psychological burden in our sample (Table 4b). “Another diagnosis, whether it is cancer or something else, pulls the rug out from under me” (ID 16). Most interviewees reported feeling helpless. Additionally, they had witnessed several losses in their families due to cancer and reported that they often dealt with grief and sadness.

During the interviews, our participants described what emotional responses of individuals with LFS they had witnessed. (Table 4c). Fear and anxiety were very prominent feelings they witnessed. These issues were related to worries about the future, worries about the results of physical examinations, intense surveillance programs, and general uncertainties due to LFS. Striking statements of the individual with LFS such as “I can't do this anymore, (…), I'm going to Switzerland and getting an injection” (Note: refers to seeking assisted suicide) (ID 26) or “We don't need to buy a camper van, I'm going to die soon anyway” (ID 6) were stuck in the heads of our interviewees. A mother of a child with LFS disclosed her shock at how LFS and the familial cancer diagnoses changed her daughter's thinking (“When she had a few bruises, she asked: Mama, is this leukemia?” ID 40).

However, fear and anxiety were not only witnessed but also experienced by our participants (Table 4d): Worries about what was going to happen when the partner gets a diagnosis or dies were often noted “Yes, sleepless nights. You think about many things. What if something happens? If something happens again, what do you do? Where do you go? How does it all end? It's all buzzing around in your head” (ID 26). Furthermore, there were financial worries, such as one husband wondering how he could or should continue working if his wife and daughter became sick. One reported thoughts about ending their own life when their child dies of cancer. The uncertainties related to LFS were seen as the “sword of Damocles” (ID 32), with negative test results from surveillance appointments lessening anxiety temporarily.

Some coping strategies on how interviewees address the psychological burdens were mentioned (Table 4e). Several times, a positive attitude toward life and optimism were perceived as beneficial (“I am optimistic, I always expect that everything will be fine again,” ID 36) and were often combined with a functional, outcome‐oriented approach (“I actually keep going, always keep going. Always one step after the other, always, no matter in what state,” ID 6). Many reported to seek distraction in their difficult daily life, for example, hobbies, sports, or spending quality time with children or partners. A few felt empowered by being activists in the LFS community.

#### 3.4.2 Main category II: health system and information needs

These statements revolved around participants' experiences within the health‐care system; often, the desire for expertise on LFS was mentioned (Table 4f). For most, finding the right health‐care professional for their partner/relative/friend with LFS was a challenging journey: “It's just the way it is with LFS; they sort according to the type of cancer. One is responsible for sarcoma, the other for carcinoma. And if you don't have an acute illness, you don't fit into the grid” (ID 7). The participants felt that it was difficult to keep track of years of documents on test results and appointments.

The participants also reported their experiences with psychosocial support offered within the health‐care system (Table 4g). The previously mentioned feelings of helplessness and uncertainty were noted as reasons for receiving psychosocial support. Thoughts such as “It worries me that I am probably not as good of a supporter as I should be” (ID 72) and questions such as “what can I do for myself?” (ID 64) were disclosed. One participant said that, as not directly affected by LFS, one is “not the victim” (ID 76), therefore having more restraints to request support. The participants reported several requirements for psychological support, such as: support being offered only if needed; long‐term supervision, especially at the beginning; support should be offered even if the diagnosis is not verified; expertise knowledge; no waiting periods; accessibility. One participant said, “it would be nice to at least be asked” (ID 6) how they felt when they accompanied their partner/relative/friend with LFS.

We identified perceived involvement in their person with LFS life as another relevant category (Table 4h). On the one hand, some of the participants were less involved: “I read that surveillance note, but I do not know much about it” (ID 6); “I only get involved when there is a result” (ID 76). On the other hand, the involvement was high, with some taking the leading role in organizing and information gathering: “Someone has to keep the overview, and that's mostly me, who always knows which things are pending and which are not” (ID 63); “Everything always goes through me and everyone also talks to me. And I just think that's good” (ID 32). They reported taking responsibility for organizing appointments, accompanying the individual with LFS as often as possible or organizing childcare or work. While this was a source of strength, some reported being overburdened and overwhelmed by this: “I wasn't ready to have to restrict my life so much and be so changed in my daily routine with everything” (ID 6). Most participants reported sharing financial struggles with their loved one, for example, entering an objection at health insurance companies for their partner.

#### 3.4.3 Main category III: communication needs

The communication in families, partnerships, and friendships was often reported to be dominated by LFS‐related topics (Table 4i): “Somehow it's all about doctors and diagnosis and diseases and, yes, it's already partly annoying” (ID 16). It was viewed as positive to share experiences and disclose feelings, but on the other hand, it was burdening. One described how everyone in the family keeps each other updated about surveillance appointments and results. A feeling of togetherness in families was reported. The connection with the partner/spouse was often noted to be closer because of LFS (“Our relationship has been influenced a little, but actually rather positively,” ID 36). However, if there were different opinions on how to handle LFS, it led to smaller and larger disputes within families and partnerships. An extremely burdensome impact of LFS on communication was when participants had to disclose adverse diagnoses to other family members, especially children.

Interviewees reported how they perceived communication with health‐care professionals, mostly physicians (Table 4j). Negative experiences were shared, such as one doctor saying, “I don't read what you send to me” (ID 40). Several times, the interviewees stated that they were not seen or taken seriously by health‐care professionals: “For the doctors, I am not the patient, I am just the affiliated” (ID 61); “As a partner, I felt completely abandoned” (ID 6). One wished to at least be noticed in the sense of “good thing you are here with her” (ID 16). Another participant stated his pressing need to be noticed by health‐care professionals: “I don't play a role there. I don't have a problem. It's not about me. (…) Nobody is interested in that at all. No one has ever asked me about it” (ID 6). Some felt that they had to compensate for the lack of expertise of health‐care professionals, for example, by researching themselves. To ease communication, remote messaging of results and information was seen as beneficial.

## 4 DISCUSSION

In this explorative mixed‐methods study on partners, relatives, and friends of individuals with LFS, we identified several uSCN mostly in terms of emotional/psychological issues and health‐care information services. We observed notably high levels of distress (70%) and fear of progression (65%) in most participants, alongside clinically low levels in mental (30% of participants) and physical (44%) health‐related quality of life. These results underscore a substantial psychological burden in caregivers of individuals with LFS.

### 4.1 Psychosocial and physical burden

A comparison of the proportions of high FoP and high distress in partners and relatives with findings from our prior study on individuals with LFS revealed few to no differences between these two cohorts (FoP: no LFS 55.8%, LFS 68.6%; distress: no LFS 69.8%, LFS 69.1%) (Kiermeier et al., 2024). This result is in line with findings from numerous studies of cancer patients and their partners, where proportions of high levels of depression and anxiety were also comparably high, and FoP in spouses was found to be even greater than that in their diseased partner (Tong et al., 2024; Wu et al., 2023). Disease perception, stress resilience, and symptoms such as the FoP of patients and loved ones are linked to each other (Tong et al., 2024). Illness perceptions and feelings of control were found to be associated with less FoP in familial caregivers (O'Rourke et al., 2021). We hypothesized that the high uncontrollability and reported feelings of helplessness associated with LFS diagnosis significantly contributed to FoP in both partners and their relatives. This is supported by “worry” and “fears” being identified as problems by a large proportion of our participants and by our qualitative data, in which fears and worries represent their own category (“It is pure fear…”, Table 4d).

Our results concerning partners' and relatives' distress and HRQOL notably exceeded the prevalence reported in a previously investigated cohort in the Netherlands (Lammens et al., 2011). This may be because recruitment for our study coincided with COVID‐19‐related social restrictions (e.g., not being allowed to accompany others to their health‐care appointments, closed childcare), which may have exacerbated the psychological burden in our cohort. Furthermore, our interviews revealed significant gaps in psychosocial support systems for relatives and partners and identified emotional and organizational obstacles that hinder their accessibility and utilization (e.g., “It's not about me”, “When I care for my sister's children during her check‐ups, it's always hard to plan. Plus, I have a family myself.”). The interviews further reveal barriers not only on the relatives' side but also on the part of healthcare providers (“no one ever asked me about it”). This suggests a mutual reinforcement of the lack of psychosocial support for caregivers, as objectively in quantitative results, our participants do, in fact, report unmet emotional and psychological needs. Surprisingly, no uSCN regarding communication and family needs were among our top 10 noted uSCN, while communication within the family and with healthcare providers has been shown to be indeed relevant in interviews (Rising et al., 2022). The confrontation with suicidal ideations mentioned in interviews is not present in our quantitative data. These indicate that the SCNS P&C might not address all the unique needs of caregivers of individuals with LFS adequately enough. Given that the take‐up of psychological support is a possible mediator of psychosocial burden, this might also contribute to the high levels of distress in our participants (Lammens et al., 2011).

Compared to our previous study, relatives, partners, and friends reported similar low scores in physical HRQOL as individuals with LFS (no LFS, 30.2%; LFS, 34.8%), as well as several physical problems, such as fatigue, pain, and sleep disturbances (Kiermeier et al., 2024). This indicates a persistent caregiver burden in relatives of individuals with LFS that manifests in psychophysiological symptoms (Litzelman et al., 2018). However, physical problems were not present in interview analysis, suggesting that caregivers might be less aware of their physical symptoms and their possible connection to psychological distress.

### 4.2 Dealing with psychosocial burden and unmet needs: Emotional and problem‐focused coping

Summarizing our qualitative and quantitative results, interestingly, we discovered “emotional and problem‐focused coping” strategies in our cohort—a known concept that has been used in research on cancer caregivers: “Emotion‐focused coping” involves regulating the emotional response to a stressful situation without trying to change the stressor itself. “Problem‐focused coping” aims to change the stressor by taking active steps to solve or alter the situation (Perez‐Ordonez et al., 2016). Our partners and relatives expressed much involvement in organizing life around LFS in interviews and often reported unmet health and information needs in SCNS P&C. This indicates a “problem‐oriented coping” strategy of partners, relatives, and friends to address the uncontrollability of LFS and the reported feelings of helplessness. Supporting their partner or relative with LFS via organization may be a way to compensate for this feeling. It appears to be a common strategy for caregivers to accumulate information in order to deal with FoP (Banks et al., 2023). This “problem‐oriented coping” can be combined with an individual's identified role of “health leader” in their LFS family (Pantaleao et al., 2020). This role comes with several responsibilities, including obtaining and disseminating new health‐related information, facilitating healthcare appointments, and serving as other members' LFS expert or advocate. Our results indicate that partners and relatives often take on this role and therefore report unmet needs to fulfill this role, which is a significant part of “problem‐oriented coping.”

What we additionally observed are “emotional‐coping strategies,” which are relevant for relatives and partners, as they revealed to have the greatest unmet need in dealing with “feelings about death” and are confronted with suicidal ideations of the individual with LFS. We found several indications that partners and relatives deal with these challenges with emotion‐focused coping: for example, optimism toward one self and the individual with LFS, being thankful for momentary good physical health or being more present in the moment (Werner‐Lin et al., 2020). In general, it seems that, particularly for LFS, high identification with the syndrome itself seems to be a central point of dealing with the condition for individuals with LFS as well as for their partners and relatives (Wilsnack et al., 2021; Young et al., 2019). This can be seen as a highly relevant “emotion‐focused coping” strategy for both groups, diagnosed individuals and caregivers. However, this coping strategy may also have disadvantages, as strong identification can act as a double‐edged sword, potentially increasing distress by heightening awareness of uncontrollability (Wilsnack et al., 2024). Our study confirms that this is also the case for friends as so‐called “voluntary kin” (Peters et al., 2016). “Dysfunctional coping” (e.g., refusing to believe the situation has happened) has also been previously described in cancer caregivers (Perez‐Ordonez et al., 2016) and we found some indications for it in our study on individuals with LFS (e.g., family members ignoring the possibility of inherited LFS) (Kiermeier et al., 2025). In this study on partners, friends, and relatives, however, we found no hints for this coping strategy. Also, in contrast to a previous study on individuals with LFS, religion or spirituality as a coping strategy was absent in our interviews (Peters et al., 2016).

### 4.3 Strengths and limitations

The perspective of partners and others, who are living through the experience of LFS with the diagnosed individual, has rarely been evaluated in psychosocial research and never, to our knowledge, in a German cohort. Our study addressed this gap with qualitative methods and validated quantitative questionnaires. However, there are several limiting factors in our study. First, our sample size was rather small, restricting our analysis options and generalizability. Nevertheless, the themes we identified are in line with those of previous studies (Barnett et al., 2022). Second, we cannot provide the attrition rate, as we do not know how many possible participants were directly or indirectly addressed. Third, we did not assess race or citizenship as variables. Fourth, our sample likely has selection bias: those who are involved in LFS and have a strong bond with their partner/relative/friend with LFS were probably more likely to take part in a study such as this one. Therefore, we were not able to gain insight into relationships in which LFS caused disruptions, as some participants reported, or whether this might have led to different results. Lastly, we adapted the wording of the validated questionnaires with the aim to match our cohort. However, we performed a reliability test, and our adapted versions revealed good to excellent internal consistency.

For this study, we were not able to conduct a matched analysis of LFS and non‐LFS partners. For future research, we will consider exploring actor–partner interdependence models such as if spouses' FoP is negatively associated with patients' HRQOL as previously found in couples with cancer patients (Sauer et al., 2022). Additionally, longitudinal data to obtain closer insights into the dynamics of these interactional effects throughout the course of LFS are needed.

### 4.4 Practical implications

Roughly two broad categories of uSCN can be identified among partners, relatives, and friends of individuals with Li‐Fraumeni syndrome (LFS): (1) organizational–practical needs, such as finding and coordinating expert information, organizing medical appointments and communication, managing childcare, and addressing financial concerns; and (2) deep–existential needs, including fears about death and dying, feelings of regret or moral dilemmas regarding having children, and coping with the emotional reactions of the diagnosed individual. A case manager or other organizational support could absorb an enormous part of the information gathering and organizational responsibility relatives and partners often described. Considering the emotional challenges, as they are not the person with an LFS diagnosis, our results revealed that opportunities for and initiation of psychosocial support are scarce and difficult. Health‐care professionals need to provide information, perhaps in the form of leaflets that exclusively address partners, relatives, or friends. This should include information on caregiver burden and options to seek psychosocial support as a non‐diagnosed person. Online psychoeducational interventions may be one way of addressing needs in partners of individuals with hereditary cancers (Tercyak et al., 2012). Additionally, health‐care professionals should not ignore the partners, relatives, or friends as when they accompany individuals with LFS. Instead, they should directly address them and ask about their mental and physical states. A short psychosocial screening, such as the distress thermometer, could be used to (1) identify the need for support (Werner‐Lin et al., 2020) and (2) serve as a means to validate the social circle (Shaffer et al., 2019). Strengthening familial resilience as a whole could be an advantageous focus for psychosocial interventions in patients with hereditary cancer syndromes such as LFS (Wilsnack et al., 2021; Young et al., 2019). However, different types of relationships (e.g., partners, siblings, friends) may need different supportive interventions (Dang et al., 2022). Additionally, age‐ and life stage‐specific interventions should be considered for both groups (those with LFS and those without LFS) (Barnett et al., 2022).

## 5 CONCLUSION

LFS along with its associated high risk for multiple cancer types impacts not only the diagnosed individual but also presents an organizational and emotional burden to their partners, relatives, and close friends. As an autosomal dominant inherited syndrome, LFS particularly affects all family members, complicating matters such as family planning and turning them into significant emotional challenges. Partners and relatives play a major role in supporting diagnosed individuals but also have their own distinct needs, challenges, and coping strategies. These may be overlooked or neglected in both personal and clinical settings. Therefore, psychosocial support that directly addresses them is essential, for example, via interventions with familial or age‐specific approaches, screening for psychosocial symptoms in partners and relatives and providing targeted information.

## AUTHOR CONTRIBUTIONS

SK: writing – original draft, data curation, formal analysis, investigation; methodology. SS: conceptualization, project administration, funding acquisition, resources, supervision, writing – review and editing. JN: writing – review and editing, investigation, data curation. CMD: data curation, writing – review and editing. FS: writing – review and editing. CPK: conceptualization, funding acquisition, project administration, resources, writing – review editing. MK: writing – review and editing, investigation. IM: conceptualization, project administration, funding acquisition, resources, supervision, writing – review and editing. The authors IM and SK confirm that they had full access to all the data in the study and take responsibility for the integrity of the data and the accuracy of the data analysis. All of the authors gave final approval for this version to be published and agree to be accountable for all aspects of the work in ensuring that questions related to the accuracy or integrity of any part of the work are appropriately investigated and resolved.

## FUNDING INFORMATION

SS, IM, CPK, and MK were supported by the Bundesministerium für Bildung und Forschung, Federal Ministry of Education and Research, BMBF (01GM1909D), CPK has been supported by the Deutsche Kinderkrebsstiftung (DKS2024.03), JN was supported by the Faculty of Medicine Heidelberg in the form of the Rahel‐Goitein‐Strauss fellowship.

## CONFLICT OF INTEREST STATEMENT

SK, SS, JN, CMD, FS, CPK, MK, and IM declare no conflicts of interest.

## ETHICS STATEMENT

Human studies and informed consent: Approval to conduct this research on human subjects was obtained from the institutional review boards of University Hospital Heidelberg and Hannover Medical School. All procedures followed were in accordance with the ethical standards of the responsible committee on human experimentation (institutional and national) and with the Declaration of Helsinki (revised 2013). Informed consent was obtained from all patients prior to their inclusion in the study.

Animal studies: No nonhuman animal studies were carried out by the authors for this article.

## Supporting information


Data S1:

## Data Availability

The data that support the findings of this study are available from the corresponding author upon reasonable request.
